# Contribution of Extracellular Membrane Vesicles To the Secretome of Staphylococcus aureus

**DOI:** 10.1128/mbio.03571-22

**Published:** 2023-02-06

**Authors:** Divakara SSM Uppu, Xiaogang Wang, Jean C. Lee

**Affiliations:** a Division of Infectious Diseases, Department of Medicine, Brigham and Women’s Hospital and Harvard Medical School, Boston, Massachusetts, USA; The University of Mississippi Medical Center

**Keywords:** *Staphylococcus aureus*, extracellular membrane vesicle, secretome, proteomics, membrane proteins

## Abstract

The microbial secretome modulates how the organism interacts with its environment. Included in the Staphylococcus aureus secretome are extracellular membrane vesicles (MVs) that consist of cytoplasmic and membrane proteins, as well as exoproteins, some cell wall-associated proteins, and glycopolymers. The extent to which MVs contribute to the diverse composition of the secretome is not understood. We performed a proteomic analysis of MVs purified from the S. aureus strain MRSA252 along with a similar analysis of the whole secretome (culture supernatant) before and after depletion of MVs. The MRSA252 secretome was comprised of 1,001 proteins, of which 667 were also present in MVs. Cell membrane-associated proteins and lipoteichoic acid in the culture supernatant were highly associated with MVs, followed by cytoplasmic and extracellular proteins. Few cell wall-associated proteins were contained in MVs, and capsular polysaccharides were found both in the secretome and MVs. When MVs were removed from the culture supernatant by ultracentrifugation, 54 of the secretome proteins were significantly depleted in abundance. Proteins packaged in MVs were characterized by an isoelectric point that was significantly higher than that of proteins excluded from MVs. Our data indicate that the generation of S. aureus MVs is a mechanism by which lipoteichoic acid, cytoplasmic, and cell membrane-associated proteins are released into the secretome.

## INTRODUCTION

Extracellular membrane vesicles (MVs), a class of lipid nanovesicles, are released by Gram-positive bacteria, such as Bacillus subtilis (1), Staphylococcus aureus (2–7), Staphylococcus epidermidis (8), Streptococcus pneumoniae (9), and Listeria monocytogenes (10). Depending on the strain characterized, the cargo of S. aureus MVs includes a variety of cytoplasmic and membrane proteins, glycopolymers, and nucleic acids (5, 7, 11–15). The generation of S. aureus MVs is dependent on the secretion of phenol-soluble modulin (PSM) peptides that alter the cytoplasmic membrane (2, 5). Moreover, MV release is enhanced by autolysins and by treatment with beta-lactam antibiotics or mutations that diminish the cross-linking of the peptidoglycan cell wall (2). S. aureus MV production is augmented by a variety of environmental stresses encountered by the bacterium during infection (12, 16, 17), and these MVs exhibit cytotoxicity to multiple cell types (2, 14, 18, 19). S. aureus MVs elicit the production of proinflammatory mediators *in vitro* and *in vivo* and induce atopic dermatitis-like inflammation in mice (4, 6, 11, 19–21), and co-administration of MVs exacerbates S. aureus infection (4). These findings indicate that MV production likely impacts the pathogenesis of staphylococcal infections.

Gram-negative bacteria carry sophisticated secretion systems (T3SS, T4SS, and T6SS) that transport proteins from the cytoplasm, through the inner and outer membranes, and across the host cell membrane in a one-step process (22). S. aureus lacks such mechanisms, relying on Tat, Sec, and the type VII protein secretion system to secrete exoproteins into the external environment (22–25), where they may be inactivated by neutralizing antibodies or enzymes with hydrolytic or proteolytic activities. Because MVs may be internalized within host cells (7, 11, 12), MV-associated cargo is delivered to host cells protected from environmental degradation or neutralization.

Cytoplasmic proteins are major components of the S. aureus secretome (26, 27); autolysins and PSMs play a role in this process since mutations in *atlA* or *psmα1-4* reduce their excretion (27, 28). Lipoproteins are also released into the S. aureus supernatant, and this process, too, is dependent on PSMs (2, 5). We hypothesized that the release of cytoplasmic and cell membrane-associated proteins, including lipoproteins, into the S. aureus secretome could, in part, be due to the generation of MVs since PSMs play a role in all three processes (2, 5, 27, 29, 30). Here, we characterize the secretome of S. aureus strain MRSA252 by performing proteomic analyses of purified MVs and cell-free culture supernatants before and after depletion of MVs. Our results indicate that a subset of proteins is packaged into MVs, and that this process is modulated by the subcellular localization and isoelectric point of individual proteins, as well as by their abundance in the secretome.

## RESULTS

S. aureus strain MRSA252 is representative of a highly prevalent epidemic EMRSA-16 clone that typified hospital-acquired methicillin-resistant S. aureus isolates in the United Kingdom in the late 1990s (31). MRSA252 belongs to multilocus sequence type 36, a member of clonal complex 30, one of four major clonal complexes that account for more than 90% of sequenced S. aureus genomes (32). Although MVs have been characterized from strains LAC (2, 5), N315, MW2 (13), and MSSA476 (4), we chose strain MRSA252 because its genome is more genetically diverse than those of most sequenced S. aureus isolates. The S. aureus MRSA252 chromosome is 2,902,619 bp in size and comprises 2,671 predicted protein-coding sequences (31).

### Proteomic analysis of MRSA252 MVs.

**FIG 1:**
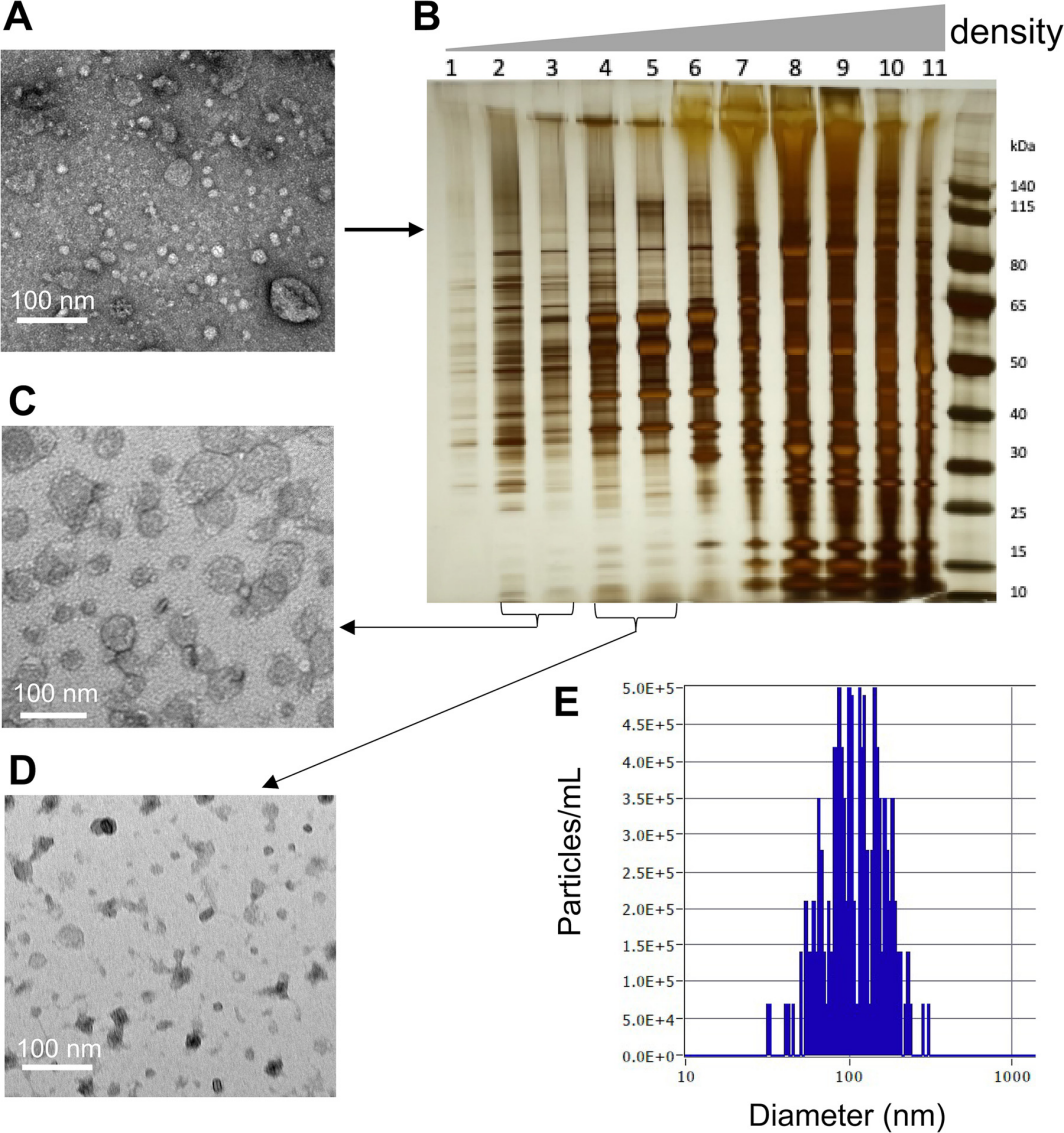
Characterization of membrane vesicles (MVs) from culture supernatants of Staphylococcus aureus MRSA252. (A) Transmission electron micrograph (TEM) of crude MVs obtained after ultracentrifugation. Crude MVs were subjected to OptiPrep density gradient ultracentrifugation to separate the MVs from membrane fragments and protein aggregates. (B) Fractions from the gradient were subjected to SDS-PAGE followed by silver staining. Fractions 2 to 3 and 4 to 5 were pooled separately and purified by size-exclusion chromatography. TEM showed purified MVs in pooled fractions 2 to 3 (C) but not in fractions 4 to 5 (D). Scale bar = 100 nm. (E) Nanoparticle tracking analysis of purified MRSA252 MVs.

**FIG 2:**
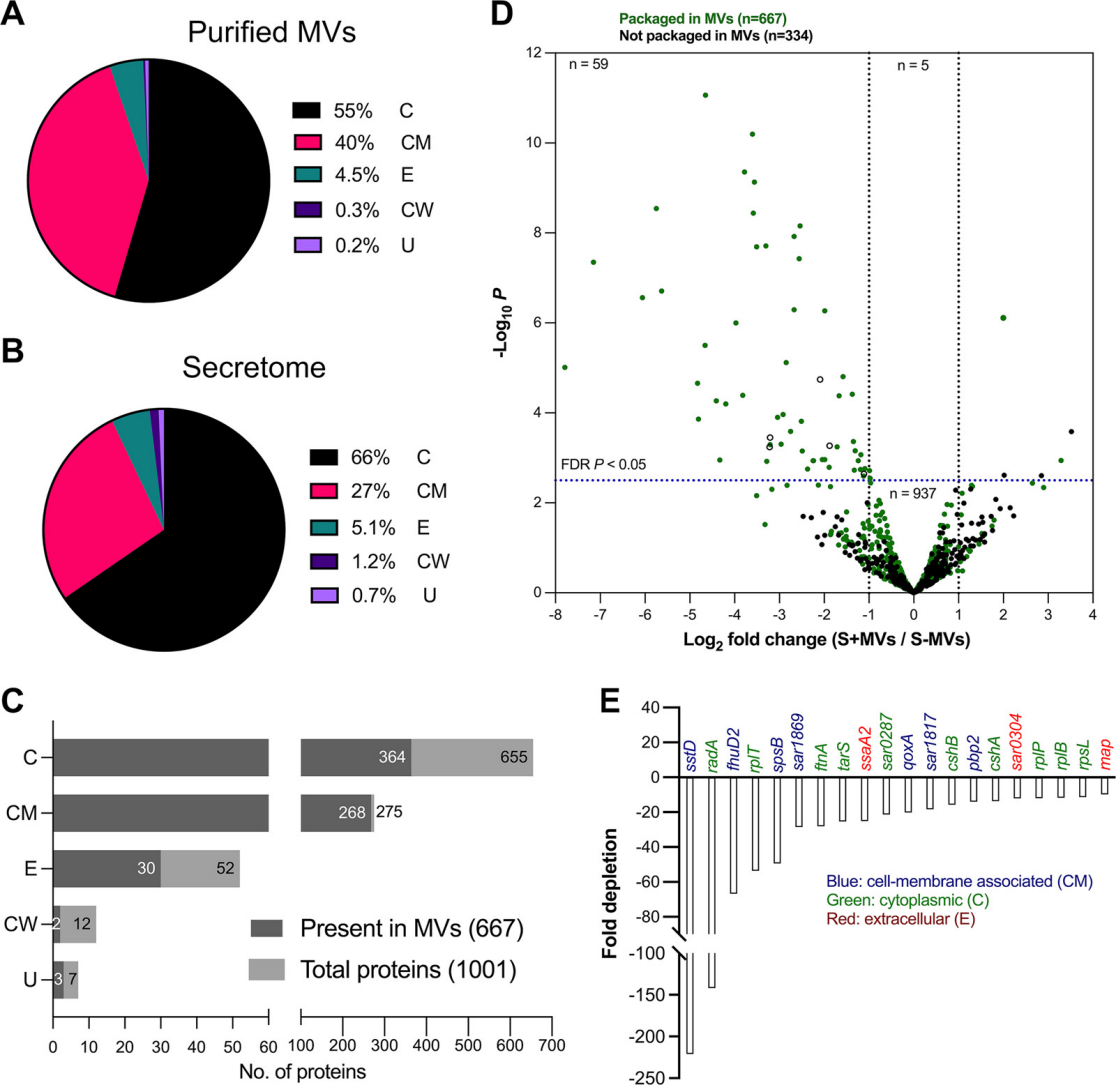
Proteomic analyses of MVs and the secretome of MRSA252. Subcellular localizations of (A) 667 MV-associated proteins and (B) 1,001 proteins in the MRSA252 secretome. (C) Subcellular localization of proteins from purified MVs within the total secretome. C, cytoplasmic; CM, cell membrane-associated; E, extracellular; and CW, cell wall-associated proteins; U, proteins of unknown localization. (D) Volcano plot of the total secretome showing significant reductions in protein abundance following depletion of MVs. Log_2_-fold change in protein intensities between culture supernatants before (S+MVs) and after (S−MVs) ultracentrifugation is plotted against the −log_10_ *P* value for 1,001 proteins in the secretome. Dotted line on the *y* axis indicates the false discovery rate (FDR)-adjusted *P* < 0.05 cutoff, whereas those on the *x* axis represent the cutoffs for depletion (log_2_-fold change <−1) or enrichment (log_2_-fold change >1) of protein intensities in the culture supernatant depleted of MVs. Significantly depleted (*n* = 59) or enriched (*n* = 5; Pgk, AirR, SdrC, Adk, and SAR1164) proteins were those with ≥2-fold change and an FDR-adjusted *P* of <0.05. After ultracentrifugation, 937 proteins in the culture supernatant showed no significant fold change in relative protein intensities. Five proteins (open black circles; BfmB, GcvPB, SAR0357, SAR2788, and SAR1672) were significantly depleted but not packaged in MVs. (E) The 20 MRSA252 proteins with a ≥10-fold depletion following ultracentrifugation of the culture supernatant to pellet MVs.

MVs were pelleted from planktonic, post-exponential culture supernatants of MRSA252 by ultracentrifugation to yield crude MVs (Fig. 1A), which were further purified by density gradient ultracentrifugation (Fig. 1B), size exclusion chromatography, and diafiltration. The structural integrity and purity of the MVs (Fig. 1C and D) was demonstrated by transmission electron microscopy (TEM). Approximately 20 μg MV protein from one liter of culture was purified from gradient fractions 2 and 3 with an average MV particle size of 104 ± 8 nm (Fig. 1E). For proteomic analyses, ~15 μg purified MVs were subjected to SDS-PAGE, and Coomassie-stained gel sections were analyzed by liquid chromatography-tandem mass spectrometry (LC-MS/MS). An analysis of purified MVs from three independent cultures of MRSA252 revealed a cargo of 667 proteins (Table S1). Subcellular localization predictions indicated that most proteins were classified as cytoplasmic (56%), followed by cell membrane-associated (40%) and extracellular (4.5%) proteins (Fig. 2A). Only 0.3% of the MV proteins were cell wall-associated, whereas the remaining 0.2% were categorized as proteins with an unknown localization (Fig. 2A). The 10 proteins most abundant in MVs (Table S1) were nine cell membrane-associated proteins (the integral membrane protein SpsB, eight lipoproteins [TycA, SAR0390, PrsA, SAR1066, SaeP, SAR2104, SAR0396, and BlaZ]), and one cytoplasmic protein (RpsK).

10.1128/mbio.03571-22.1 TABLE S1 The 667 proteins detected in purified MRSA252 membrane vesicles (MVs), listed in order of abundance. Download Table S1, XLSX file, 0.1 MB. Copyright © 2023 Uppu et al. 2023 Uppu et al. https://creativecommons.org/licenses/by/4.0/ This content is distributed under the terms of the Creative Commons Attribution 4.0 International license.

### Proteomic analysis of the MRSA252 secretome.

The proteins in the filter-sterilized culture supernatants were concentrated by adsorption onto StrataClean resin and analyzed by LC-MS/MS. Proteomic analysis of the secretome revealed a total of 1,001 proteins (Table S2). An overall view of the subcellular localization predictions of the secretome proteins (Fig. 2B) revealed a remarkable similarity to that of MVs. Cytoplasmic proteins accounted for 66% of the secretome proteins, 27% were cell membrane-associated, 5% were extracellular, 1.2% were cell wall-associated, and 0.8% were proteins with an unknown distribution. The 10 most abundant proteins in the secretome included the bifunctional autolysin AtlA, eight cytoplasmic proteins (elongation factor Tu, l-lactase dehydrogenase 1, elongation factor G, ribosomal proteins RpsB, RplK, RplQ, and RplE, and glyceraldehyde-3-phosphate dehydrogenase 1), and protein A (Spa), a cell wall-associated protein that is released into the supernatant during bacterial growth (33).

10.1128/mbio.03571-22.2 TABLE S2 The 1,001 proteins detected in S+MVs and S−MVs, listed in order of S+MV abundance. Download Table S2, XLSX file, 0.4 MB. Copyright © 2023 Uppu et al. 2023 Uppu et al. https://creativecommons.org/licenses/by/4.0/ This content is distributed under the terms of the Creative Commons Attribution 4.0 International license.

### A comparison of the MRSA252 genome, secretome, and MV proteome.

When the protein cargo of the total secretome of MRSA252 and its MVs were compared, we found that 667 of the total 1,001 secretome proteins were detected in MVs (Fig. 2C). The MRSA252 genome encodes 1303 cytoplasmic proteins; the secretome included 655 of these, and 364 were packaged in MVs (Fig. 2C). The genome encodes 700 cell membrane-associated proteins (including lipoproteins); 275 of these were detected in the secretome, and 268 were packaged in MVs. Out of a total of 507 transmembrane (TM) proteins with multiple TM domains identified in the genome, 147 were detected in the secretome, and 146 were packaged in MVs (Table S2). Of the 57 lipoproteins in the genome, 42 were detected in the secretome, and all 42 lipoproteins were packaged in MVs (Table S3). The genome encodes 89 extracellular proteins; 52 of these were detected in the secretome, and 30 were packaged in MVs. The genome encodes 35 cell wall-associated proteins; the secretome included 12 of these, but only two (SasF and Spa) were packaged in MVs. The genome has 544 proteins with an unknown subcellular localization; only 7 of these were present in the secretome, and 3 were packaged in MVs.

10.1128/mbio.03571-22.3 TABLE S3 The 42 lipoproteins identified in purified MRSA252 MVs. Download Table S3, XLSX file, 0.02 MB. Copyright © 2023 Uppu et al. 2023 Uppu et al. https://creativecommons.org/licenses/by/4.0/ This content is distributed under the terms of the Creative Commons Attribution 4.0 International license.

Proteomic analyses of filter-sterilized culture supernatants were performed before (S+MVs) and after depletion of MVs (S−MVs) by ultracentrifugation. We recovered total protein amounts of 112 ± 26 μg for S+MVs and 96 ± 22 μg for S−MVs from the same supernatant volume (Fig. S1). LC-MS/MS analysis was performed on 56 ± 13 μg protein for S+MVs and 48 ± 11 μg protein for S−MVs. Before calculating the fold change in the intensities of each protein within the S+MV and S−MV samples, the data were normalized to yield the same median log_2_ protein intensity, allowing us to quantify the reduction in protein abundance in the secretome depleted of MVs. As shown in the volcano plot (Fig. 2D), 59 proteins were significantly depleted in the culture supernatants lacking MVs, and 54 of these were present in the MVs. These preferentially MV-packaged proteins (Table 1) were comprised of 34 cytoplasmic, 10 cell membrane-associated, and 10 extracellular proteins. Based on their phylogenetic classification, MV proteins were categorized according to the Clusters of Orthologous Groups database (Table 1). Most of the 54 depleted proteins were associated with bacterial translation, ribosomal structure, and biogenesis, followed by proteins with unknown function.

**TABLE 1:** The 54 MV-associated proteins that were significantly depleted from the MRSA252 supernatant by ultracentrifugation^*a*^

Protein	Gene	kDa	Location	pI	COG classification
Poly(ribitol-phosphate) beta-*N*-acetylglucosaminyltransferase	*tarS*	66.3	C	6.2	Carbohydrate transport and metabolism
LCP family protein, SAR2394	*lcpC*	34.7	CM	9.1	Cell wall/membrane/envelope biogenesis
Penicillin-binding protein 2	*pbp2*	79.3	CM	8.6	Cell wall/membrane/envelope biogenesis
Immunoglobulin-binding protein	*sbi*	50.2	E	9.4	Cell wall/membrane/envelope biogenesis
*N*-acetylmuramoyl-l-alanine amidase	*sle1*	36	E	9.7	Cell wall/membrane/envelope biogenesis
Aerobic glycerol-3-phosphate dehydrogenase	*glpD*	62.4	C	6.4	Energy production and conversion
Probable malate:quinone oxidoreductase 2	*mqo2*	56	C	6.1	Energy production and conversion
Pyruvate dehydrogenase E1 component subunit alpha	*pdhA*	41.4	C	4.9	Energy production and conversion
Pyruvate dehydrogenase E1 component subunit beta	*pdhB*	35.2	C	4.7	Energy production and conversion
Pyruvate dehydrogenase complex E2 component	*pdhC*	46.4	C	4.9	Energy production and conversion
Probable quinol oxidase subunit 2	*qoxA*	41.8	CM	8.7	Energy production and conversion
Fibrinogen-binding protein	*ecb*	12.1	E	10.4	Function unknown
Extracellular matrix protein-binding adhesin Emp	*emp*	38.4	E	9.9	Function unknown
Extracellular adherence protein Eap/Map	*map*	76.8	E	10.0	Function unknown
Hypothetical protein	*sar0287*	60.9	C	9.7	Function unknown
5′-nucleotidase	*sar0304*	33.3	E	9.5	Function unknown
Putative exported protein	*sar0694*	10.4	E	10.1	Function unknown
Hypothetical protein	*sar1817*	18	CM	6.9	Function unknown
NERD domain-containing protein	*sar1869*	35	CM	6.3	Function unknown
Staphylococcal secretory antigen A2	*ssaA2*	29.6	E	9.0	Function unknown
Iron transporter substrate-binding lipoprotein	*fhuD2*	34	CM	9.2	Inorganic ion transport and metabolism
Bacterial non-heme ferritin	*ftnA*	19.6	C	4.6	Inorganic ion transport and metabolism
Manganese transporter substrate-binding lipoprotein	*mntC*	34.7	CM	8.7	Inorganic ion transport and metabolism
Siderophore ABC transporter substrate-binding protein	*sstD*	37.8	CM	9.3	Inorganic ion transport and metabolism
Signal peptidase IB	*spsB*	21.7	CM	9.0	Intracellular trafficking, secretion, vesicular transport
Delta-hemolysin	*hld*	2.98	E	8.2	Not available
Putative exported protein	*sar0622*	18.6	E	9.2	Not available
Uncharacterized lipoprotein SAR2457	*sar2457*	23.3	CM	6.1	Not available
Pyruvate oxidase; Thiamine pyrophosphate enzyme	*cidC*	63.8	C	7.2	Nucleotide transport and metabolism
DEAD-box ATP-dependent RNA helicase	*cshA*	56.9	C	9.5	Replication, recombination, and repair
DEAD-box ATP dependent DNA helicase	*cshB*	51	C	9.5	Replication, recombination, and repair
DNA repair protein	*radA*	49.8	C	6.6	Replication, recombination, and repair
DNA topoisomerase 4 subunit B	*parE*	74.4	C	6.5	Transcription
DNA topoisomerase 1	*topA*	79	C	9.1	Transcription
DNA topoisomerase 3	*topB*	81.5	C	9.5	Transcription
Bifunctional phosphopantothenoylcysteine decarboxylase	*coaBC*	44.1	C	5.7	Translation, ribosomal structure, and biogenesis
rRNA adenine N-6-methyltransferase	*ermA*	28.4	C	9.9	Translation, ribosomal structure, and biogenesis
Ribonuclease R	*rnr*	90.4	C	6.3	Translation, ribosomal structure, and biogenesis
50S ribosomal proteins					
L1	*rplA*	24.5	C	8.8	Translation, ribosomal structure, and biogenesis
L2	*rplB*	30.1	C	10.8	Translation, ribosomal structure, and biogenesis
L4	*rplD*	22.5	C	9.9	Translation, ribosomal structure, and biogenesis
L15	*rplO*	15.6	C	10.3	Translation, ribosomal structure, and biogenesis
L16	*rplP*	16.2	C	10.6	Translation, ribosomal structure, and biogenesis
L19	*rplS*	13.4	C	11.5	Translation, ribosomal structure, and biogenesis
L20	*rplT*	13.7	C	11.3	Translation, ribosomal structure, and biogenesis
L21	*rplU*	11.3	C	9.8	Translation, ribosomal structure, and biogenesis
L28	*rpmB*	6.97	C	12.2	Translation, ribosomal structure, and biogenesis
L35	*rpmI*	7.69	C	12.3	Translation, ribosomal structure, and biogenesis
30S ribosomal proteins					
S3	*rpsC*	24.1	C	9.8	Translation, ribosomal structure, and biogenesis
S4	*rpsD*	23	C	10.0	Translation, ribosomal structure, and biogenesis
S11	*rpsK*	13.9	C	11.2	Translation, ribosomal structure, and biogenesis
S12	*rpsL*	15.3	C	11.3	Translation, ribosomal structure, and biogenesis
S13	*rpsM*	13.7	C	10.4	Translation, ribosomal structure, and biogenesis
Probable tRNA sulfurtransferase	*thiI*	46.3	C	6.5	Translation, ribosomal structure, and biogenesis

a C, cytoplasmic; CM, cell membrane-associated; E, extracellular; COG, Clusters of Orthologous Groups.

10.1128/mbio.03571-22.5 FIG S1 Protein content of culture supernatants with (S+MVs) and without membrane vesicles (S−MVs). Filter-sterilized S+MVs and S−MVs were enriched for protein with StrataClean beads, and the protein content was determined with a bicinchoninic acid assay kit. Data are presented as mean ± standard error of the mean and analyzed with the Student’s *t* test (ns, not significant). Download FIG S1, TIF file, 0.1 MB. Copyright © 2023 Uppu et al. 2023 Uppu et al. https://creativecommons.org/licenses/by/4.0/ This content is distributed under the terms of the Creative Commons Attribution 4.0 International license.

The 20 proteins with ≥10-fold depletion in S−MVs included 10 cytoplasmic, seven cell membrane-associated, and three extracellular proteins (Fig. 2E). Although the majority (*n* = 942) of the secretome proteins were not significantly depleted by ultracentrifugation, many of these (*n* = 613) were present as MV cargo. Proteins not packaged in MVs (*n* = 334; black dots in Fig. 2D) were not significantly depleted in the S−MVs samples. Five proteins (open black circles in the top left panel of Fig. 2D) were significantly depleted but not packaged in MVs. Each of these five proteins was detected in one of three MV biological replicates, and their depletion levels were <10-fold. SAR2788 is an extracellular protein, whereas the other four (BfmB, GcvPB, SAR0357, and SAR1672) are cytoplasmic proteins. Five proteins were significantly enriched in the culture supernatant after ultracentrifugation (green and black dots in the top right panel of Fig. 2D). Two of these (cytoplasmic proteins Pgk and Adk) were packaged in MVs, whereas the other three (two cytoplasmic [AirR and SAR1164] and the cell wall-associated protein SdrC) were excluded.

**FIG 3:**
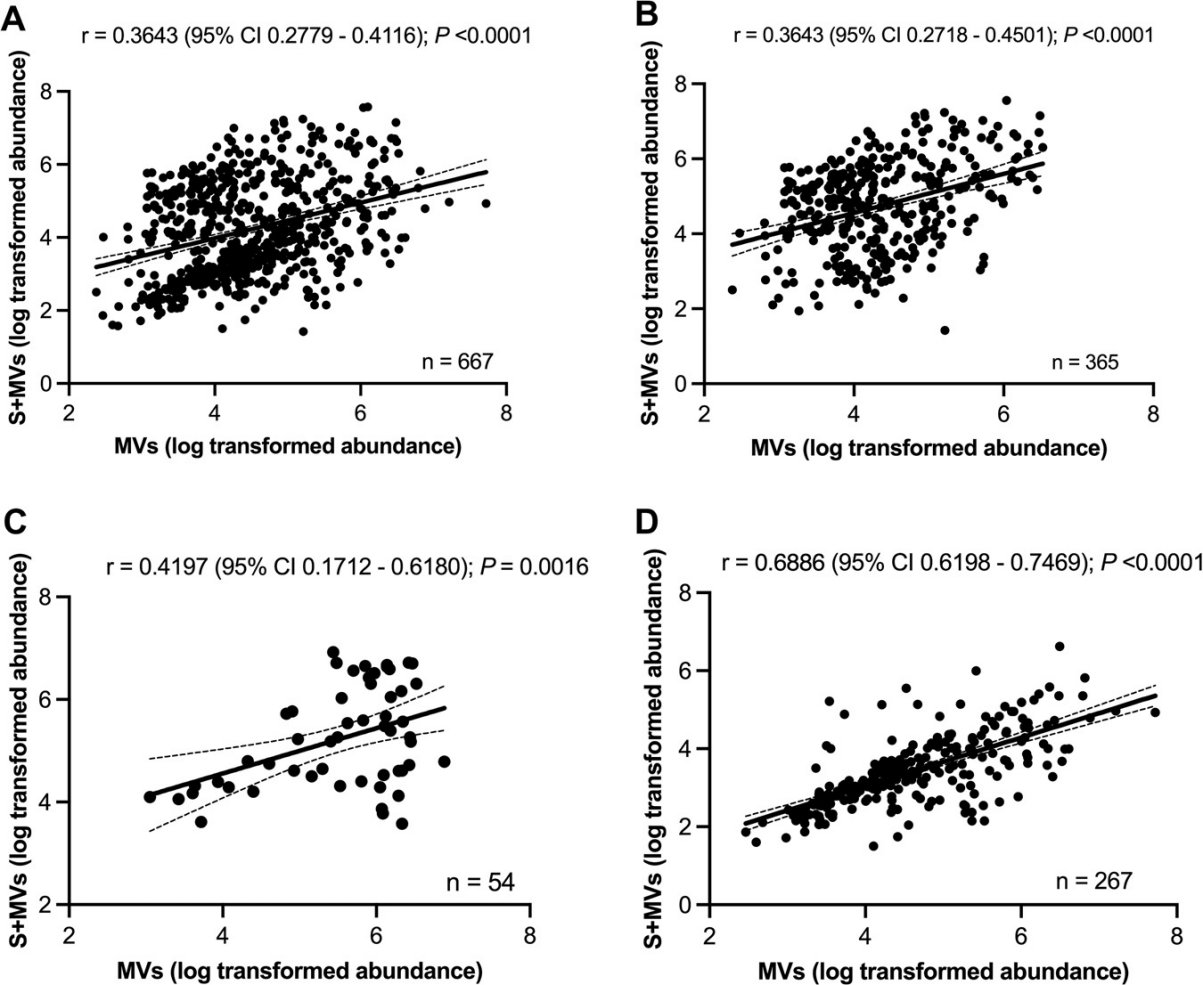
Correlation between the relative abundance of proteins in the MRSA252 secretome (S+MVs) versus the relative protein abundance in purified MVs. Graphs show the correlations between protein abundance in the secretome versus (A) all 667 proteins packaged into MVs. (B) MV-associated cytoplasmic proteins. (C) 54 proteins specifically sorted to MVs. (D) MV-associated cell membrane proteins. Distributions are shown with linear regression lines and 95% confidence intervals (CI).

We hypothesized that the relative abundance of a protein in the bacterial secretome dictates its packaging and abundance within MVs. To address this, we performed a correlation analysis of the relative abundance of individual proteins in the secretome and MVs (Fig. 3). The correlation coefficients were similar for all MV proteins (Fig. 3A), MV-associated cytoplasmic proteins (Fig. 3B), and the 54 specifically packaged MV proteins (Fig. 3C). The highest correlation was observed for the MV-associated cell membrane proteins (*n* = 267), whose abundance in the membrane-rich MVs correlated well with their abundance in the secretome (Fig. 3D). The correlations between the relative abundance of extracellular proteins (*n* = 30) and ribosomal proteins between MVs and S+MVs were not significant (not shown).

### pI of proteins in the secretome and MVs.

**FIG 4:**
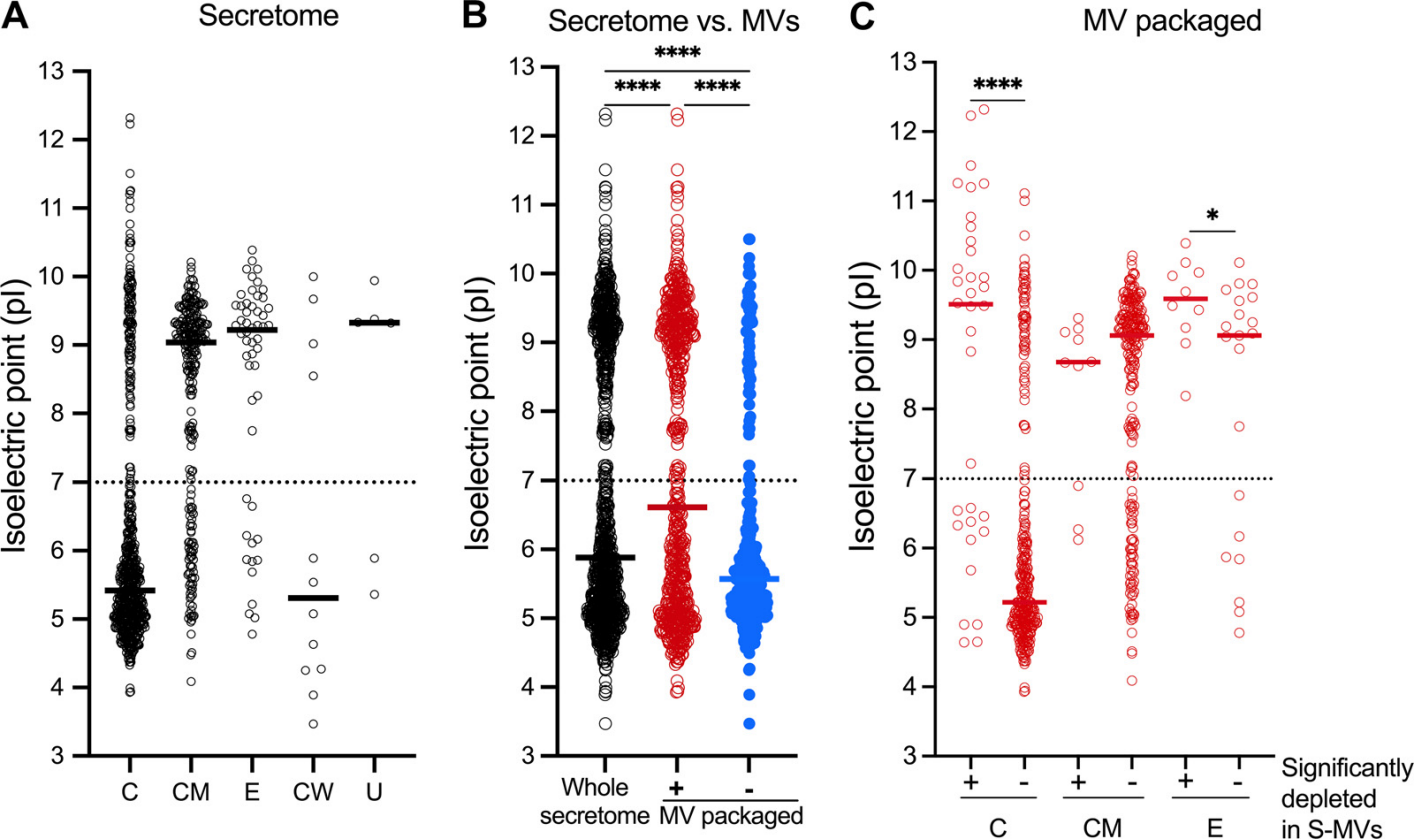
Comparison of predicted pI of proteins in the MRSA252 secretome and purified MVs. (A) pI values of secretome proteins with different subcellular localizations. (B) Distribution of pI values for the whole-secretome proteins (*n* = 1,001) and proteins either packaged (*n* = 667) or not packaged (*n* = 334) in MVs. (C) The pI values of the MV cargo proteins that are significantly depleted (+) from the culture supernatant (*n* = 54) by ultracentrifugation were compared to pI values of proteins not (−) significantly depleted (*n* = 613). Data are presented as medians; *n* = 3. Data in panels B and C were analyzed by two-way analysis of variance and the Mann-Whitney test, respectively. *, *P* < 0.05; ****, *P* < 0.0001.

Tartaglia et al. first reported that the average pI and the net charge at pH 7 of proteins packaged into S. aureus MVs differed significantly from those of the whole-cell proteome (13). To expand upon these observations, we compared the pI of proteins in the secretome with that of MV-packaged proteins. Cytoplasmic and cell wall-anchored proteins in the secretome had a median pI of ~5.5, whereas proteins that were cell membrane-associated, extracellular, or of unknown localization had a median pI of ≥9 (Fig. 4A). The pI of the secretome proteins showed a wide distribution, ranging from 3.5 to 12.5, with a median pI of 5.9 (Fig. 4B). The MV cargo proteins showed a similar pI distribution but with a significantly higher median of 6.7. Notably, most proteins excluded from MVs clustered within an acidic pI range and showed a significantly lower median pI of 5.6 (Fig. 4B). Among the 54 MV-associated proteins whose abundance in the secretome was significantly depleted by ultracentrifugation, cytoplasmic and extracellular proteins showed a significantly higher median pI compared to those MV-packaged proteins that were not significantly depleted in S−MVs (Fig. 4C). MV-packaged cell membrane proteins with or without depletion in S−MVs had pI values that were >8.5 (Fig. 4C). Overall, these data indicate that proteins packaged in MVs were enriched for those with a basic pI compared to those not packaged in MVs.

### Analysis of proteins and glycopolymers by immunoblots.

**FIG 5:**
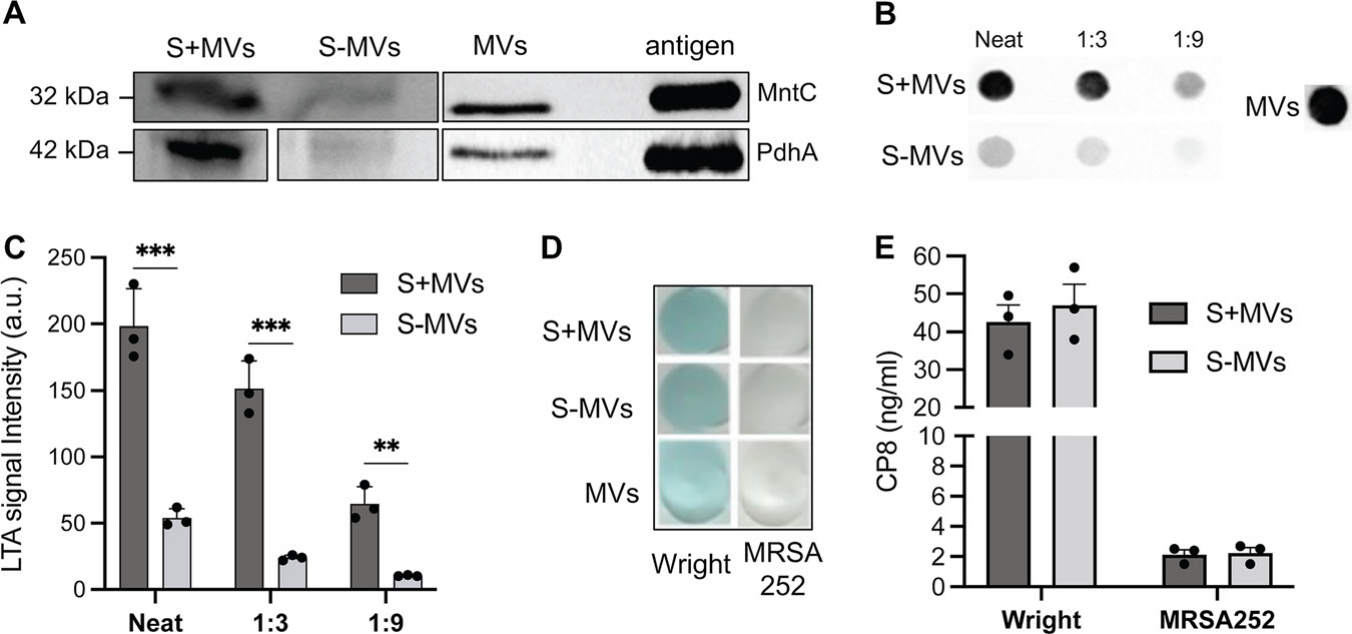
Immunodetection of MRSA252 proteins and glycopolymers in MVs and culture supernatants. (A) Immunoblot detection of proteins present in culture supernatants before (S+MV) and after (S−MV) ultracentrifugation. Purified MVs and recombinant antigens as controls were probed with mouse antiserum to the manganese transporter C (MntC) or pyruvate dehydrogenase A (PdhA). (B) Representative dot immunoblots of lipoteichoic acid (LTA) in S+MVs, S−MVs, and MVs (5 μg) detected with monoclonal antibodies to LTA. (C) Quantitation of LTA signal intensities from triplicate immunoblots. (D) Capsular polysaccharide type 8 (CP8) in the supernatant samples and in purified MVs (250 ng) was detected by dot immunoblots. (E) CP8 concentrations in S+MVs and S-MVs were quantified by ELISA. Data are presented as mean ± standard error of the mean and analyzed with the Student’s *t* test. ***, *P* < 0.001; **, *P* < 0.01. Blots are spliced for the purpose of labeling and removing the empty space between the lanes.

To support our LC-MS/MS data, we utilized immunoblots to assess the relative concentrations of selected antigens that were highly abundant in culture supernatants and MVs: the lipoprotein MntC, the cytosolic protein PdhA, and lipoteichoic acid (LTA). As shown in Fig. 5A, a reduction in the band intensities in S−MVs compared to S+MVs was observed for PdhA and MntC; both proteins were detected in purified MVs. LTA, tethered to the bacterial membrane (34), was detectable in MVs and the culture supernatant, but the band intensities were markedly reduced when MVs were depleted by ultracentrifugation (Fig. 5B). Quantitative analysis of the LTA signal intensity showed that LTA was significantly reduced by ≥75% in the S−MV samples (Fig. 5C).

S. aureus sheds capsular polysaccharides (CPs) into culture supernatants (35), and CPs were detected in MVs from strains Newman and 6850 (2). To determine whether CPs shed into the culture supernatant were primarily MV-associated, we probed dot immunoblots of S+MVs, S−MVs, and purified MVs with antibodies specific to the serotype 8 CP (CP8). Because MRSA252 produces little cell-associated or shed CP8 (36), no signal was detected in the supernatants or MVs prepared from this strain (Fig. 5D). S. aureus strain Wright produces abundant CP8 which is shed into the culture supernatant (36). Immunoblots showed a positive signal for strain Wright MVs and similar intensities between S+MV and S−MV samples (Fig. 5D). CP8 concentrations in each sample were quantified by enzyme-linked immunosorbent assay (ELISA), and no differences between S+MV and S−MV samples for either MRSA252 or Wright were observed (Fig. 5E). These findings indicate that CP8 is associated with S. aureus MVs, but soluble CP8 is also present in culture supernatants depleted of MVs.

## DISCUSSION

Our proteomic analyses showed 1,001 proteins in the MRSA252 secretome; cytoplasmic (66%) and membrane-associated (27%) proteins accounted for the bulk of the proteins. Although the mechanism(s) by which cytoplasmic proteins are released from the bacterial cell is not fully understood, autolysins and cell wall remodeling at the septal site during growth appear to play a role in this process because an *atl* mutant showed significantly fewer excreted cytoplasmic proteins compared to the wild-type strain (26). Atl was the most abundant, and *N*-acetylmuramoyl-l-alanine amidase (SAR2723; another peptidoglycan hydrolase) was among the 10 most abundant proteins in the MRSA252 secretome. Cytoplasmic protein excretion has also been attributed to the production of PSMα peptides (37), which alter the bacterial cell membrane due to their surfactant-like characteristics (27).

Of the 1,001 proteins in the secretome, 667 were packaged in MVs, and the protein composition of MVs mirrored that of the secretome. Notably, 268/275 (97%) of S. aureus cell membrane-associated secretome proteins were packaged in MVs, presumably because the bacterial membrane is pinched off during MV biogenesis (2). All 42 lipoproteins in the secretome were packaged in MVs, and 5 of them (SstD, FhuD2, MntC, SAR2457, and QoxA) were significantly depleted in S−MVs. Among the 10 most abundant MV proteins, 8 are lipoproteins (Table S1), indicating their high abundance in membrane-rich MVs. We detected 52 exoproteins in the secretome, and 30 (58%) were contained within the MV cargo. Packaging of exoproteins in MVs likely occurs randomly as the peptides are transported by traditional mechanisms through the bacterial membrane, consistent with our observation that the abundance of exoproteins in the secretome and MVs showed a significant correlation. We also demonstrated that ~75% of LTA detected in the culture supernatant was MV-associated, likely because LTA itself is membrane-anchored. In contrast, CP8 is thought to be linked to the cell wall (38, 39), and it was detected equally in MVs as well as in soluble form in the culture supernatants.

Proteomic analyses of MVs from multiple S. aureus strains, including LAC (5), JE2 (2), MW2 (13), MSSA476 (4), and several clinical isolates of human (15) or bovine origin (13, 14, 18) have been reported. There exists a common core MV proteome of 119 proteins, which is primarily comprised of cytoplasmic and membrane-associated proteins (13), and we found that 106 of these 119 core proteins were present in MRSA252 MVs (Table S4). Four of the missing 13 proteins were absent from the MRSA252 genome. Due to genetic variability among different S. aureus isolates, the MV proteome often contains strain-specific features (13). The type of culture medium, incubation temperature, and duration of cultivation affect MV protein cargo (4, 5, 40). Staphylococcal enterotoxin A is produced by both MRSA252 and MSSA476, but this superantigen is only packaged in MSSA476 MVs (4, 31). MRSA252 MV cargo included fewer pore-forming toxins than other isolates (12); leukocidin AB, gamma-hemolysins, and delta hemolysin (Hld) are components of the MRSA252 secretome, but the MV cargo only included Hld. Likewise, the proteases aureolysin (Aur) and staphopain A (ScpA) are detected in the MRSA252 secretome, but only ScpA was packaged in MVs. The overall protein yields for strain MRSA252 MVs were lower than that of LAC (2), possibly due to decreased *agr* expression by MRSA252, resulting in reduced secretion of PSMs (41).

10.1128/mbio.03571-22.4 TABLE S4 The 119 core proteins identified in S. aureus MVs. Download Table S4, XLSX file, 0.02 MB. Copyright © 2023 Uppu et al. 2023 Uppu et al. https://creativecommons.org/licenses/by/4.0/ This content is distributed under the terms of the Creative Commons Attribution 4.0 International license.

Basic proteins in the MRSA252 secretome were enriched in its MVs. Tartaglia et al. first reported selective packaging of positively charged bacterial proteins in S. aureus MVs (13). We observed that MV-packaged proteins had an overall higher average pI value compared to staphylococcal proteins that were in the secretome but excluded from MVs. The 5 proteins that were significantly enriched in the supernatant after MV depletion had acidic pI values between 4 and 5.5. Of the 54 proteins that were significantly depleted from the MRSA252 secretome by pelleting MVs, 38 were basic in nature, including 15 ribosomal proteins with a pI of ≥10.5. Negatively charged domains of the bacterial membrane could promote electrostatic interactions with these cationic proteins at sites of MV formation. Ribosomal proteins are abundant in S. aureus culture supernatants (26–29) and account for 12 out of 119 core MV proteins (13). During protein synthesis, nascent cell membrane proteins are targeted to the Sec translocase in the bacterial membrane by the signal recognition particle (42), allowing for proper insertion of the polypeptides into the membrane. This process results in a functional association of ribosomes translating membrane proteins with the cytoplasmic membrane (43), a process that could contribute to the enrichment of ribosomal proteins in MVs during their biogenesis.

### Conclusions.

A proteomic analysis of MVs generated by the hospital-acquired epidemic S. aureus strain MRSA252 showed that its cargo consisted primarily of cytoplasmic, cell membrane-associated, and extracellular proteins. The proteome of the culture supernatant (secretome) of MRSA252 was analyzed before and after ultracentrifugation to remove MVs. The secretome comprised 1,001 proteins, 667 of which were also present in MVs. The compositions of the secretome and MVs were strikingly similar and led to the identification of 54 specifically MV-packaged proteins. The pI and subcellular localization of proteins, as well as their abundance in the secretome, influenced the preferential packaging of MV proteins. Our findings provide evidence that the generation of S. aureus MVs is a mechanism by which lipoteichoic acid, cytoplasmic, and cell membrane proteins are excreted into the secretome.

## MATERIALS AND METHODS

### Bacterial strains.

S. aureus strain MRSA252 (NRS71) was obtained from BEI Resources. S. aureus strain Wright is ATCC 49525 (44).

### Isolation and purification of MVs.

MRSA252 was cultivated with shaking at 37°C in tryptic soy broth to an OD_650_ (optical density at 650 nm) of 1.2. MVs were purified as described previously with a few modifications (2). Filter-sterilized culture supernatants were concentrated 25-fold by tangential flow filtration with a 100-kDa polyethersulfone membrane system (Centramate, Pall Corp.). The concentrated supernatants were ultracentrifuged at 150,000 × g at 4°C for 3 h to pellet crude MVs, which were gently suspended in phosphate-buffered saline (PBS) and observed by TEM. To remove membrane fragments and protein aggregates, the crude MVs were purified by density gradient ultracentrifugation in 40% to 15% OptiPrep medium (density = 1.215 to 1.085 g/mL). After ultracentrifugation at 140,000 × g for 16 h at 4°C, aliquots of 1 mL gradient fractions were analyzed by SDS-PAGE and silver staining. Fractions 2 to 3 and 4 to 5 were pooled, and the two samples were purified over Sepharose CL-6B resin (Cytiva) to remove the OptiPrep medium. Fractions (0.5 mL) were collected and monitored for protein at *A*_280_ (absorption at 280 nm) and OptiPrep medium at *A*_244_. After different fractions were visualized by TEM, those enriched for MVs were pooled and diafiltered with centrifugal filters (10 kDa, polyethersulfone, Thermo Fisher Scientific) at 3,500 × g for 15 min at 4°C. Purified MVs were filter-sterilized, and protein concentrations were determined with Bio-Rad protein dye. MVs were stored at −80°C until further use.

### Enrichment of proteins from culture supernatants.

MRSA252 was cultivated as described above. Bacteria were centrifuged at 8,000 × g for 30 min at 4°C, and the filter-sterilized (0.45 μm) supernatant was split into two equal 25-mL samples. One sample was stored at 4°C, while the second sample was centrifuged at 150,000 × g at 4°C for 3 h to pellet the MVs. The resulting supernatant and the untreated sample were each mixed with 20 μL of activated StrataClean beads (Agilent) and incubated overnight with rotation at 4°C. The beads were activated by treatment with 12 M hydrochloric acid at 100°C for ≥6 h and washed with Tris-EDTA buffer before use. The protein-enriched beads were sedimented at 13,500 × g at 4°C, washed with Tris-EDTA buffer, and dried with a SpeedVac Concentrator. The protein-enriched beads were rehydrated in 20 μL PBS, and their protein content was determined with a bicinchoninic acid assay kit (Thermo Fisher Scientific).

### Proteomic analysis by LC-MS/MS.

The protein-enriched StrataClean beads were rehydrated with 20 μL freshly prepared loading buffer as described previously (45). After denaturing the proteins at 98°C for 10 min, 10-μL samples were electrophoresed in MOPS (morpholinepropanesulfonic acid) buffer at 120 V on a 4 to 12% Bis-Tris NuPAGE gel. After washing, the gels were placed in a fixing solution (10% acetic acid and 40% ethanol) for 30 min, washed, and stained with Coomassie blue. Excised gel bands were cut into ~1-mm^3^ pieces, subjected to a modified in-gel trypsin digestion procedure (46), and analyzed by LC-MS/MS using electrospray ionization and an LTQ Orbitrap Velos Pro ion-trap mass spectrometer (Thermo Fisher Scientific). Peptides were detected, isolated, and fragmented to produce a tandem mass spectrum of specific fragment ions for each peptide. For S+MV, S−MV, and MV samples, three biological replicates for each sample were analyzed by LC-MS/MS. Peptide sequences (and hence protein identities) were determined by matching the NCBIProt database of the MRSA252 genome with the acquired fragmentation pattern by the software program SEQUEST (47) (Thermo Fisher Scientific). All databases include a reversed version of all the sequences (decoy database search). To remove contaminants and reverse hits, data were filtered to maintain an average 0.8% and 0.1% false discovery rate for protein and peptide identifications, respectively. Protein intensity was quantified as the summation of intensity values (peak height intensity) of individual peptides matched to a given protein. For enhanced confidence in protein identification, individual proteins in each data set (S+MVs, S−MVs, and MVs) were further filtered to maintain a minimum requirement of two total peptides and one unique peptide in at least two of the three biological replicates.

Bioinformatic analyses were performed using the R package (v4.1.3). Protein intensities were log_2_-transformed to achieve an approximately normal distribution of the data. We found that higher-abundance proteins had fewer missing values, indicating that missing values were at least partially due to low abundance. Since some proteins were present in only two samples, we input missing values using half the minimum intensity per biological replicate. Finally, we normalized the values in each of the three independent experiments such that all data sets had the same median log_2_ protein intensity. To discover proteins which showed differential abundance between the paired S+MV and S−MV samples, we determined the fold change based on the normalized log_2_ protein intensities. Statistical analyses were done using the R package *limma* (48), which performs linear regression modeling and moderated *t* tests (two-sided). For protein differences with *P* < 0.05, we calculated the false discovery rate (49) and applied a threshold of 0.05. Proteins were annotated with gene names using the MRSA252 genome from the Uniprot, KEGG, and AureoWiki databases. Subcellular localization of the proteins was predicted by PSORTb v3.0.3, CELLO (50), and AureoWiki (51), and theoretical pI values were computed by Expasy (Uniprot). TM proteins were identified using the TMHMM-2.0 web-based TM domain prediction tool (52). Correlations in relative protein abundance between MVs and S+MVs were evaluated using Spearman’s *r*.

### Transmission electron microscopy.

Purified MVs were adsorbed onto Formvar/carbon coated copper grids and negatively stained with 1% uranyl acetate. The samples were imaged on a JEOL1200EX electron microscope (JEOL, Peabody, MA) equipped with an AMT 2k CCD camera (Advanced Microscopy Techniques Corp., Danvers, MA).

### Nanoparticle tracking analysis.

Nanoparticle tracking analysis was performed with a Zetaview QUATT Particle Tracking Analyzer (Particle Metrix). The instrument was calibrated with 100-nm polystyrene beads before measurement of purified MVs (total protein concentration 20 μg/mL) at 25°C. The settings included an average of 11 different positions for quantification of MV size.

### Western blots for protein detection.

MVs or 10 μL of protein-enriched StrataClean beads were subjected to SDS-PAGE and transferred to PVDF (polyvinylidene difluoride) membranes. Recombinant His-MntC and His-PdhA were used as controls. The membranes were blocked for 1 h with PBS + 5% skim milk and washed with PBS + 0.05% Tween 20 (PBST). The membranes were incubated overnight at 4°C with mouse polyclonal antiserum specific for MntC or PdhA (1:500 or 1:1,000 in PBS + 5% skim milk) followed by washing with PBST. The membranes were incubated for 1 h at room temperature with horseradish peroxidase (HRP)-conjugated goat anti-mouse IgG (1:5,000) in PBST + 5% skim milk. After washing with PBST, membranes were developed with a chemiluminescent HRP substrate and imaged with an iBright FL1500 instrument.

### Detection of glycopolymers by dot immunoblots.

MVs or serial dilutions of S+MV and S−MV samples were applied to nitrocellulose membranes using a 96-well Bio-dot apparatus. After washing with PBS, the membranes were treated with trypsin (4 mg/mL in 10 mM phosphate buffer [pH 8]) for 2 h at 37°C to digest Spa and Sbi (53). After blocking and washing, the membranes were incubated with 1 μg/mL of CP8 antibodies (rabbit polyclonal) or LTA mouse monoclonal antibodies (IBT Bioservices). After washing and incubation with the corresponding HRP-conjugated secondary antibodies (1:10,000 to 1:15,000), CP8 membranes were developed with TMB substrate, and LTA membranes with a chemiluminescent HRP substrate. The signal intensity of dot blot images was quantified by Image J.

### Capture ELISA.

CP8 shedding was quantified as described previously (36). Filter-sterilized S+MV and S−MV samples were boiled for 10 min to denature proteins and inactivate proteases. Next, 96-well plates (Maxisorp) were coated overnight at 4°C with 1.5 μg/mL CP8-specific monoclonal antibody 5A6 in 0.05 M sodium carbonate-bicarbonate buffer (pH 9.6). The plates were washed with PBST and blocked with 0.05% skim milk for 1 h. After washing, 3-fold serial dilutions of the boiled supernatants or purified CP8 were incubated overnight at 4°C on the coated plates. After washing, the captured CP8 was detected with a rabbit polyclonal CP8 antiserum (1:30,000). Following a 2-h incubation and washing, HRP-conjugated goat anti-rabbit IgG (1:5,000) was added. After 2 h, the plate was washed and TMB (3,3′,5,5′-tetramethylbenzidine) peroxidase substrate was added. After stopping the reaction with acid, we recorded the absorbance values at 450 nm. The data were analyzed using a five-parameter logistic equation; a purified CP8 standard curve was used to calculate CP8 concentrations in the samples.

### Statistical analysis.

Data were analyzed using GraphPad Prism (v9.3.1 Mac OS). Statistical analyses were performed using the Student’s *t* test, Mann-Whitney test, or two-way analysis of variance (Tukey’s multiple-comparison test). *P* < 0.05 was considered significant.

### Data availability.

The mass spectrometry proteomics data were deposited to the ProteomeXchange Consortium via the PRIDE (54) partner repository with the data set identifier PXD035662.
