# Supplementary figures and images for: Contribution of Extracellular Membrane Vesicles To the Secretome of Staphylococcus aureus
Source: mBio. 2023 Feb 6;14(1):e03571-22. doi: 10.1128/mbio.03571-22 (PMC9973311; doi:10.1128/mbio.03571-22)

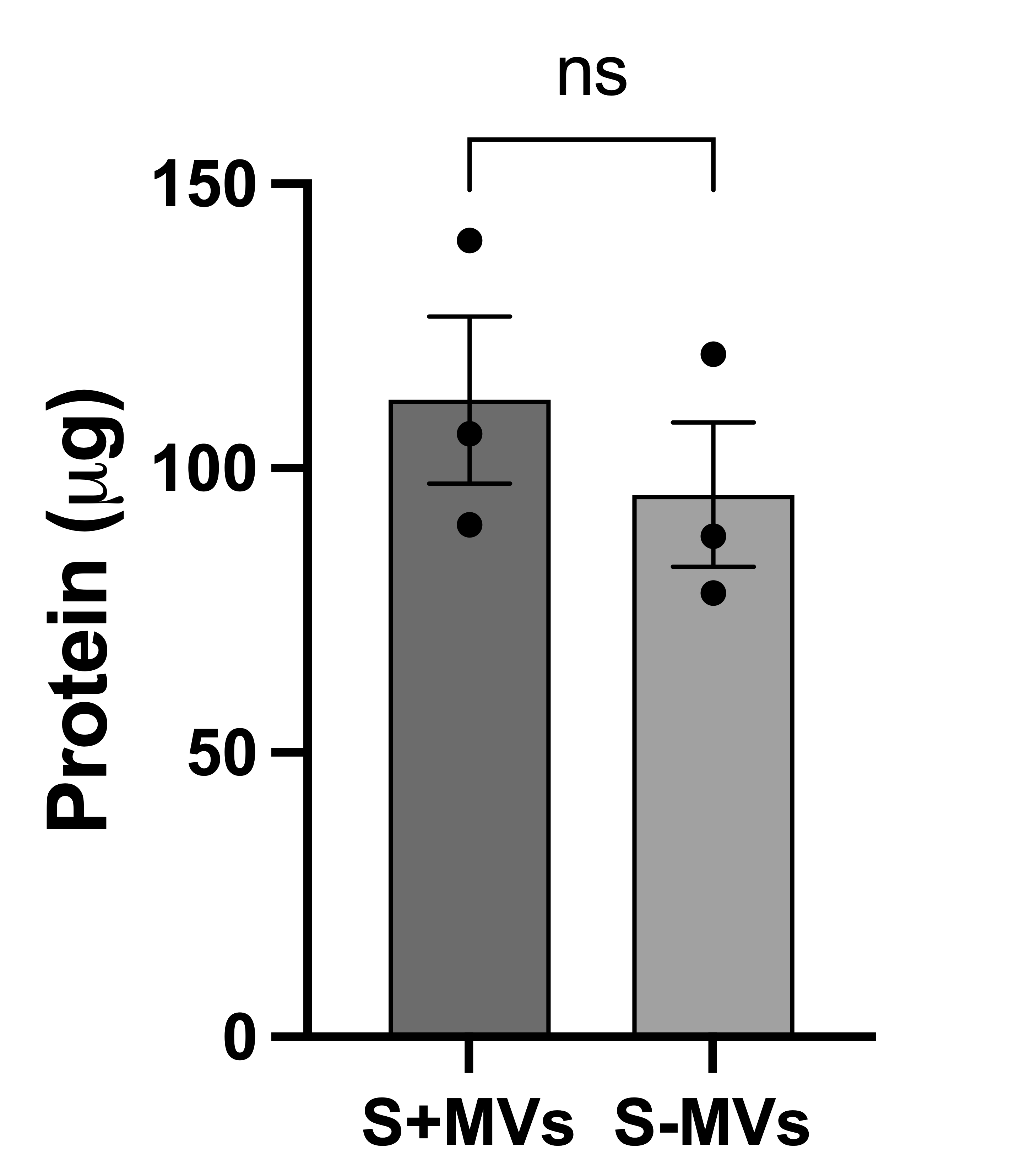

Supplement: FIG S1 [file mbio.03571-22-s0001.tif]
